# Immune Thrombocytopenic Purpura (ITP) as an Uncommon Extraintestinal Complication of Crohn's Disease: Case Vignette and Systematic Literature Review

**DOI:** 10.1155/2020/4785759

**Published:** 2020-03-26

**Authors:** Raisa Epistola, Tiffanie Do, Ritika Vankina, Daniel Wu, James Yeh, Michael W. Fleischman, Jennifer M. Lee

**Affiliations:** ^1^Harbor-UCLA Medical Center, Department of Medicine, Torrance, CA, USA; ^2^Harbor-UCLA Medical Center, Department of Medicine, Division of Hematology & Medical Oncology, Torrance, CA, USA; ^3^Harbor-UCLA Medical Center, Department of Medicine, Division of Gastroenterology, Torrance, CA, USA; ^4^University of California Los Angeles Division of Digestive Diseases, Los Angeles, CA, USA

## Abstract

While the association of immune thrombocytopenic purpura (ITP) and inflammatory bowel disease (IBD) has been described in a few case reports, management of ITP as an extraintestinal manifestation of Crohn's disease (CD) is less studied. There are approximately a dozen cases describing the management of patients dually diagnosed with CD/ITP. Previous reports postulated that the mechanism of ITP in CD was through the presence of circulating immune complexes in the serum and antigenic mimicry due to increased mucosal permeability in active colitis, versus increased mucosal production of TH1-type proinflammatory cytokines during CD flares, which may account for remission of ITP with surgery for CD. We present a case of a 27-year-old man who presented with medically refractory CD and ITP who responded to surgical management with colectomy and splenectomy, along with a systematic review of the literature. These cases suggest that colectomy should be considered in the treatment of medically refractory ITP among patients with concomitant CD.

## 1. Introduction

Immune thrombocytopenic purpura (ITP) is characterized by the presence of autoantibodies against platelet surface antigens leading to immune-mediated platelet destruction. While the occurrence of ITP and inflammatory bowel disease (IBD), including Crohn's disease (CD) and ulcerative colitis (UC), has been described in a limited number of case reports, the association of ITP as an extraintestinal manifestation of Crohn's disease has been sparsely reported [1]. Chronic inflammation, chronic intestinal bleeding, iron malabsorption, impaired dietary intake, and inadequate erythropoietin production contribute to anemia as a frequent finding in CD patients [2]. The humoral and cellular immune mechanisms that contribute to the onset of IBD suggest the association with ITP is not coincidental. Since the first reported cases of ITP associated with Crohn's disease in the late 1980s, there have been 13 case reports that have described treatment of ITP in Crohn's disease [3–5]. We present a case that queries the treatment choice for ITP in a patient with active Crohn's disease flare through postulated immune mechanisms.

## 2. Case Description

A 27-year-old man with a history of CD and ITP lost to follow-up for two years presented with two to three days of dark, bloody diarrhea, nausea, and nonbloody, nonbilious vomiting. Physical exam was notable for mild periumbilical tenderness; no hepatosplenomegaly was present.

The patient had been diagnosed with Crohn's disease seven years prior to this admission and had known moderate to severe colonic disease. He was diagnosed with ITP during a Crohn's disease flare four years prior to this presentation, was treated with IV methylprednisolone, and discharged on a prednisone taper. The direct antiglobulin test (Coombs) was negative; d-dimer, fibrinogen, C3, and C4 levels were within normal limits. Serologic studies for ANA, ANCA, viral hepatitis panel, and HIV were also negative. He did not undergo a bone marrow biopsy at time of ITP diagnosis.

**Figure 1:**
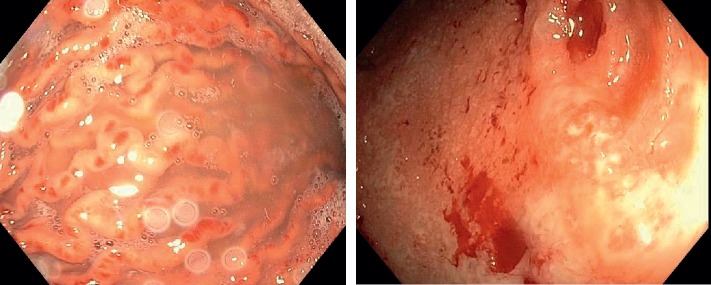
Left panel: upper endoscopy showing gastropathy. Right panel: flexible sigmoidoscopy showing moderate to severe proctosigmoiditis, Mayo Score-3.

**Figure 2:**
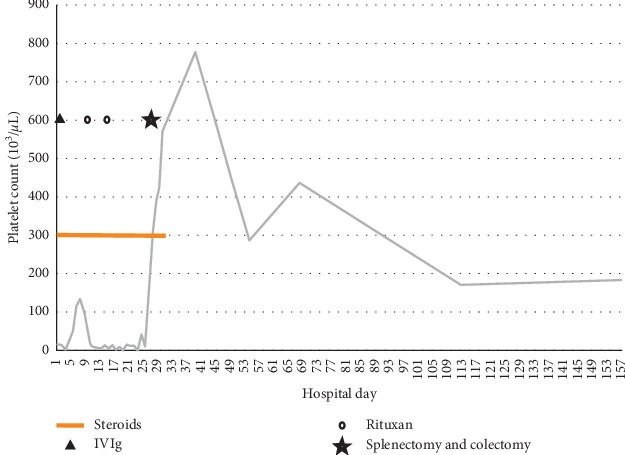
Platelet count trend and treatments. The patient was treated initially with steroids from day 1 to day 29 with concurrent IVIG on day 3 and 4 where there was a slight increase in platelets. Rituximab was given on day 10 and 17 with no drastic change in platelet count. Following splenectomy and colectomy on day 25, the platelet count increased without further treatment or platelet transfusions. The patient was discharged on day 29 without steroids.

On admission, laboratory results were notable for platelets of 18,000 per microliter (normal 160,000–360,000 per microliter), ESR of 49 mm/hr (normal 0.00–12 mm/hr), and CRP of 8.04 mg/L (normal 0.00–0.74 mg/L). He was started on methylprednisolone 20 mg IV every 8 hours and given 2 units of platelets, with platelet count improvement to 51,000 per microliter. He subsequently received intravenous immunoglobulin (IVIG) 1 g/kg daily for two days. He continued to experience bloody diarrhea and underwent esophagogastroduodenoscopy and flexible sigmoidoscopy, which revealed significant body and fundus gastropathy as well as moderate to severe proctosigmoiditis (Figure 1). He had a prior history of thrombocytopenia with adalimumab and infliximab, so he received a 390 mg IV loading dose of ustekinumab for his Crohn's disease flare. Antibody testing for anti-infliximab was negative during this hospitalization. His platelet count briefly responded to combined corticosteroids and IVIG but subsequently declined (Figure 2). He received rituximab on days 10 and 17 but had persistent thrombocytopenia. On hospital day 24, CRP increased to 5.99 mm/hr (from 0.09 mm/hr on hospital day 15), and symptoms for colitis persisted. Given his transfusion-dependent thrombocytopenia refractory to medical therapy, the patient underwent splenectomy. Due to persistent symptoms of bloody diarrhea and cramping, the patient concurrently underwent total colectomy with end ileostomy.

Following surgery, the patient's platelet count improved without transfusion support to 571,000 per microliter by the date of discharge. Pathologic review of the resected colon demonstrated changes consistent with chronic inflammatory disease, with severe disease activity. Five months following discharge, he has been maintained on ustekinumab and 6-mercaptopurine and has not had a Crohn's disease flare. His platelets remained stable at 170,000 per microliter without additional medical management for his ITP.

## 3. Discussion

Immune thrombocytopenic purpura may present as an extraintestinal manifestation of Crohn's disease. While there are at least 40 case reports describing the association of ITP and ulcerative colitis (UC) in the literature [6], there are fewer reports of dual diagnoses among Crohn's patients, particularly adults. While the prevalence of ITP among patients with ulcerative colitis is estimated at 0.1%–0.48%, the prevalence of patients with ITP and Crohn's disease is not known [8]. Given the relative dearth of information on the association of Crohn's disease and ITP, we performed a comprehensive literature search of the Medline database between March–May 2019. Keywords used included “Crohn's disease,” “idiopathic thrombocytopenic purpura,” “immune thrombocytopenic purpura,” and “ITP.” Titles and abstracts were reviewed to identify cases. References of included articles were reviewed to identify additional cases. The literature search identified 12 case reports of concomitant Crohn's disease and ITP and 1 small case-control study of IBD patients with autoimmune cytopenias, including ITP (Table 1).

Of the 13 case reports identified in our search, 12 cases demonstrated coexisting ITP and CD flare, suggesting that the two disease processes run in parallel. The parallel occurrence of these two processes was also seen in the one case-control study (*n* = 40 cases with coexisting IBD and autoimmune cytopenias; *n* = 160 controls with IBD alone) which reported 64% of IBD patients who presented with concomitant ITP and IBD flares. Compared to controls with IBD alone, there was a significantly higher frequency of extraintestinal manifestations and increased likelihood for severe acute flares among dual diagnosis patients, further supporting that autoimmune cytopenias such as ITP may be an extraintestinal manifestation of IBD [1].

Although the precise pathophysiology of concomitant ITP and Crohn's disease remains unclear, previous literature postulated the mechanism of ITP in Crohn's disease through the presence of circulating immune complexes in the serum and antigenic mimicry due to increased mucosal permeability in active colitis [8, 9]. Platelet surface antigens may carry similar peptide sequences as bacterial glycoproteins found in the gut; inflammation during IBD flare increases exposure to these antigens, resulting in antigenic cross-reactivity [10]. All but two cases identified in our literature review presented without colonic involvement. A preponderance of colonic involvement of CD has also been reported in the pediatric literature, suggesting that inflammation of the colon and/or dysregulation of colonic immunity contributes to the pathogenesis of CD-associated ITP [17, 18]. Bowel surgery could result in decreased exposure to cross-reactive antigens, and thus reduced ITP activity. Another postulated mechanism is mucosal production of TH1-type proinflammatory cytokines (IL-2 and IFN-gamma), which have been shown to be elevated in patients with ITP. Colectomy may reduce the production of these cytokines and thus reduce platelet destruction [18, 19].

The 2019 American Society of Hematology (ASH) guidelines on management of immune thrombocytopenic purpura recommend first-line therapy with corticosteroids, IVIG, or anti-D immunoglobulin as they have the quickest initial response [20]. Despite treatment with both corticosteroids and IVIG, our patient had no response and continued to be transfusion-dependent. In a typical patient with ITP unresponsive to initial corticosteroid therapy, ASH guidelines recommend thrombopoietin receptor agonists (TPO-RA), rituximab, and splenectomy as subsequent treatment options [20].

Our patient had persistent bloody diarrhea, abdominal pain, and transfusion-dependent thrombocytopenia for 24 days despite maximal medical therapy for CD. Because his CD did not improve over this prolonged period, he was offered a colectomy, as per the American College of Gastroenterology's (ACG) guidelines for medically refractory CD [21]. The patient expressed a desire to control his illnesses with minimal long-term medications, so he was offered a splenectomy over TPO-RA therapy. He underwent both colectomy and splenectomy on hospital day 24. After surgery, the patient's hematochezia resolved, as did his thrombocytopenia, and he did not require further platelet transfusions after surgery.

Our literature review identified one case of ITP that was refractory to steroids and briefly responded to splenectomy; five months after splenectomy, the ITP recurred with a CD flare. The patient then underwent colectomy, after which his thrombocytopenia resolved [5]. Another case report documented medically refractory ITP that responded to bowel resection alone [16].

A case-control study of patients with IBD and autoimmune cytopenias included 25 patients with both IBD and ITP. This group comprised of 13 patients (52%) with a diagnosis of CD and 12 patients (48%) with a diagnosis of UC. Of these 25 patients with ITP, 15 were given steroids and 7 (46.7%) achieved a complete response with steroids alone. 4 (16%) received rituximab as second-line therapy, but these patients did not have a complete response. There were also six cases of IBD and autoimmune cytopenia (four of whom had ITP) who received rituximab. Of this group, four of six showed some response to rituximab; however, the only patient that achieved a complete response had a dual diagnosis of CD and autoimmune hemolytic anemia, not ITP. In the three cases with IBD and ITP treated with infliximab, a complete response was achieved for both disease processes. This study also reported two cases with IBD/ITP who underwent radical surgery (ileocecal resection and total colectomy) and achieved remission of ITP, though it is not reported whether these two cases received steroids or rituximab prior to surgery. Although this case-control study demonstrated varying responses to rituximab and infliximab, the small sample size queries the efficacy of nonsteroidal medical therapies in dual diagnosis patients [1].

The studies described above also bring to light the uncertainty of optimal surgical management of medically refractory ITP in the setting of Crohn's disease flare. In this context, our case queries further management of additional colectomy/bowel resection in cases of ITP during active Crohn's disease unresponsive to medical therapy.

ITP is a rare, but known, extraintestinal manifestation of CD. Treatment of ITP-associated Crohn's disease can be managed medically with steroids, IVIG, and biologics (i.e., rituximab and infliximab); however, our case adds to the small body of evidence suggesting the efficacy of colonic resection as part of definitive treatment of ITP associated with Crohn's disease.

## Figures and Tables

**Table 1:** Case reports of Crohn's disease and immune thrombocytopenic purpura.

Title	Sex	Age	Interval between diagnoses	Concomitant CD and ITP flare	Location of Crohn's disease	Treatment (medical)	Treatment (surgical)	Outcome
Kosmo et al [5]	F	65	35 years	Yes	Colon	Steroids	Splenectomy, resection of rectal stump with terminal ileostomy	Refractory to steroids, resolved with surgical treatment

Manzano et al [7]	M	54	Simultaneous	Yes	Pancolitis with ileal involvement	Steroids	None	Resolved

Arruda et al [3]	M	43	2 weeks	Yes	Colon	Steroids	None	Resolved

Baudard et al [4]	F	19	Simultaneous	Yes	Duodenum, ileum, colon	IV steroids, IVIG	Splenectomy	CD symptoms improved; ITP refractory to medical and surgical treatment, patient lost to follow-up

Boyne and Dye [8]	F	17	9 years	Yes	Pancolitis without ileal involvement	IVIG, cyclosporine, steroids	None	Resolution of both ITP and CD symptoms

Selby et al [9]	M	15	5 months	Yes	Proximal colon	IV steroids, IVIG	None	ITP resolved with some residual CD symptoms on discharge

Kuloğlu et al [10]	M	5	1.5 years	No	Gastroesophageal, sigmoid	Steroids, IVIG	Splenectomy	CD responded to steroids, ITP medically refractory requiring splenectomy

Tsibouris et al [11]	M	44	Simultaneous	Yes	Ileocecal, sigmoid	IV steroids with azathioprine maintenance for Crohn's disease	None	Resolution

Germandis et al [12]	F	69	18 months	Yes	Ileocolonic	IVIG, steroids, ciprofloxacin, metronidazole, azathioprine, infliximab	None	Both CD and ITP symptoms resolved with infliximab, refractory to other therapies

De Rossi et al [13]	M	41	10 years	Yes	Ileum	IV steroids, ciprofloxacin, metonidazole infliximab	Surgical fistula sanitation and protective ileostomy	Anaphylaxis to infliximab; resolution of ITP and Crohn's disease following both medical and surgical treatment

Shaaban and Maroules [14]	M	57	Simultaneous	Yes	Gastric	IV steroids with mesalamine maintenance for Crohn's disease	None	Resolution

El Rassy et al [15] (2015)	F	25	Not reported	Yes	Ileocecal	Steroids, IVIG, rituximab, eltrombopag, methotrexate	Splenectomy	Resolution of ITP with eltrombopag, incomplete control of CD symptoms on methotrexate

Mizuno et al [16]	M	32	18 years	Yes	Ileocecal	IVIG, steroids, infliximab, azathioprine	Ileocecal resection	CD & ITP medically refractory, but both resolved with surgical treatment
